# Decreasing the Rate of Surgical Site Infection in Patients Operated by Cesarean Section in a Tertiary Care Hospital in India: A Quality Improvement Initiative

**DOI:** 10.7759/cureus.34439

**Published:** 2023-01-31

**Authors:** Tamkin Khan, Enas Mushtaq, Fatima Khan, Ayesha Ahmad, K Aparna Sharma

**Affiliations:** 1 Obstetrics and Gynecology, Jawaharlal Nehru Medical College, Aligarh, IND; 2 Obstetrics and Gynecology, Himalayan Institute of Medical Sciences, Swami Rama Himalayan University (SRHU), Dehradun, IND; 3 Microbiology, Jawaharlal Nehru Medical College, Aligarh, IND; 4 Obstetrics and Gynecology, Era’s Lucknow Medical College and Hospital, Lucknow, IND; 5 Obstetrics and Gynecology, All India Institute of Medical Sciences, New Delhi, Delhi, IND

**Keywords:** pdsa cycles, peri-operative antibiotic prophylaxis, cesarean section, surgical site infection(ssi), quality improvement projects

## Abstract

Background

Surgical site infections (SSIs) are a substantial cause of maternal morbidity and are associated with a significant increase in hospital stay and cost. The prevention of SSI is complex and requires the integration of a range of measures before, during, and after surgery. Jawaharlal Nehru Medical College (JNMC), Aligarh Muslim University (AMU) is one of the referral centers of India with a huge influx of patients.

Methods

The project was undertaken by the Department of Obstetrics and Gynaecology, JNMC, AMU, Aligarh. Our department was sensitized to the need for quality improvement (QI) through Laqshya, a Government of India initiative for labor rooms in 2018. We were facing problems like a high surgical site infection rate, poor documentation and records, no standard protocols, overcrowding, and no admission discharge policy. There was a high rate of surgical site infections, leading to maternal morbidity, increased days of hospitalization, more usage of antibiotics, and increased financial burden. A multidisciplinary quality improvement (QI) team was formed comprising obstetricians and gynecologists, the hospital infection control team, the head of the neonatology unit, staff nurses, and multitasking staff (MTS) workers.

Results

The baseline data were collected for a period of one month and it was found that the rate of SSI was around 30%. Our aim was to decrease the rate of SSI from 30% to less than 5% over a period of six months. The QI team worked meticulously, implemented evidence-based measures, regularly analyzed the results, and devised measures to overcome the obstacles. The point-of-care improvement (POCQI) model was adopted for the project. The rate of SSI decreased significantly in our patients and the rates are around 5% persistently.

Conclusion

The project not only helped in decreasing the infection rates but also led to vast improvements in the department with the formulation of an antibiotic policy, surgical safety checklist, and admission-discharge policy.

## Introduction

Jawaharlal Nehru Medical College and Hospital (JNMCH), Aligarh Muslim University, is one of the tertiary care hospitals of India with an annual delivery rate of 7000-7500. Out of these, 60-75% are booked cases. The cesarean section (CS) rate is 40-50%, as we are a tertiary care center and cater to high-risk cases, many of whom are referred to us from peripheral areas in emergencies. About five to 15 CS, four to five minor cases, and 2-4 laparotomies are performed per day on average. We have two operation theaters for clean cases and one for infected cases with no separate minor operation theaters. With a huge influx of patients and a very high surgical load, the SSI rate was very high in our facility, increasing the morbidity, hospital stay, and financial burden to the facility and patients.

Baseline data were collected from the postoperative ward for a period of one month, and it was found that the rate of surgical site infection (SSI) in patients who had undergone cesarean section was around 30%.

Aim statement

Our aim was to decrease the rate of SSI in the patients operated on by cesarean section from 30% to less than 5% over a period of six months at JNMC, AMU, India.

Background

The Centers for Disease Control and Prevention (CDC) defines SSI as an infection of the surgical site within 30 days of the operative procedure. It may be superficial, involving skin and subcutaneous tissues, deep incisional SSI involving the fascial and muscle layers, or organ/space SSI [1]. There is a wide variation in the reported infection rates, depending on the risk factors in the population, hospital practices, the definition of SSI used, the number of days of patient follow-up and hospital stay, or the surveillance applied. The universal use of the CDC definition of SSI can remove these disparities [2-4].

SSIs are among the most preventable healthcare-associated infections and are a substantial burden to healthcare systems and service payers worldwide in terms of patient morbidity, mortality, and increased hospital stay, leading to overcrowding and additional costs [5]. The emergence of antimicrobial-resistant pathogens increases the cost and challenge of treating SSIs [6]. Following a systematic review of the literature and meta-analyses, the WHO reported in 2010 that the prevalence of healthcare-associated infections in low-income and middle-income countries (LMICs) was two to 20 times higher than in high-income countries [7,8]. SSI was the most surveyed and most frequent healthcare-associated infection in LMICs, affecting up to a third of patients who underwent surgery [7,8]. The GlobalSurg Collaborative reported an overall SSI incidence of 12.3%. There was wide variation between countries: about 9.4% in high-HDI countries and 23.2% in low-HDI countries (P <.001) [9].

Cesarean section is the commonest major surgical operation performed all over the world. The majority of the CS performed are emergency cases where patients are more likely to get SSIs. There has been a rising trend in CS rates all over the world. This has increased maternal morbidity and mortality as CS is associated with a five-fold to 20-fold increase in the risk of infection compared with vaginal delivery [10]. The incidence of SSI after CS was reported to be between 3% and 24% with lower rates in HICs (high-income countries) and higher in LMICs (lower and middle-income countries) [11-13]. However, a recent study by Medecins sans Frontieres in four sub-Saharan African countries shows results of SSI after CS can be comparable to those in HIC if evidence-based standardized protocols, peri-operative antibiotics, instrument sterilization, and incision care are followed in low-resource settings improving the overall quality of care [14]. It has been estimated that approximately half of the SSIs are preventable by the application of evidence-based strategies [15].

This article was previously presented as a poster at RCOG Virtual World Congress 2021.

## Materials and methods

Measurement

To make our data collection easy, we decided to include only superficial and deep SSI as per CDC definition in our study in the initial phase. Surgical site infection was defined as purulent discharge from a wound and/or persistent fever ≥100° F for ≥48 hours after the first 24 hours of surgery till 30 days.

SSI rates per 100 CS procedures were calculated by dividing the number of SSIs by the number of total CS procedures and multiplying the results by 100 (Table 1).

**Table 1:** Measurement of the rate of SSI in the patients undergoing CS CS: cesarean section

INDICATOR	Number of patients with SSI
Numerator	SSI in the number of patients undergoing CS within 1 month of surgery
Denominator	Total number of patients operated by CS
Frequency of data analysis	Monthly
Data sources	Maternity OT nurse in-charge and surgical ward nurse in-charge records

We utilized two important QI tools to solve the problem after brainstorming with the QI team.

Duration of the study

The duration of the study was six months.

Analysis of the problem

**Figure 1:**
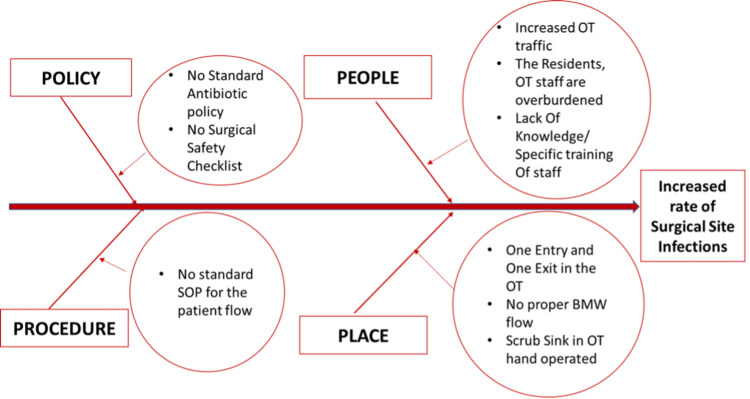
Root cause analysis (fishbone analysis)

A fishbone analysis was done (Figure 1). There were problems at multiple levels, which led to increased chances of infection. The main problems identified were no standard protocols, no surgical safety checklist, and no standard antibiotic policy. The residents and staff were overburdened because of the increased workload and long duty hours but more importantly, there was a lack of knowledge and specific training for the staff. There was no proper flow of biomedical waste (BMW) in the operation theater (OT). There was increased OT traffic because of the lack of designation and distribution of duties and responsibilities among the staff members. Being the tertiary referral center with a huge influx of patients and no standard admission-discharge policy, there was overcrowding in the wards, which not only increased the workload of the staff but also predisposed to increased infection rates. The scrub sinks in OT were hand-operated which increased the chances of infection.

Study design

A multidisciplinary quality improvement (QI) team was formed comprising obstetricians and gynecologists (OBG), the hospital infection control committee (HICC), the head of the neonatology unit, the nurse in-charge labor room, and the nurse in-charge obstetric operation theater, one staff nurse, and multitasking staff (MTS) worker each from the labor room and the operation theater.

The QI team used the point-of-care quality improvement (POCQI) model, which has been designed by the WHO to build capacity for quality improvement in health facilities by a team of healthcare workers. The unique feature and strength of the POCQI model are that it offers a simplified common sense approach that has been tested successfully and helps bring incremental improvement in the quality of care within the available resources and without many additional resources. Workshops were conducted in the hospital and the staff was trained for using POCQI intervention in the QI projects.

**Figure 2:**
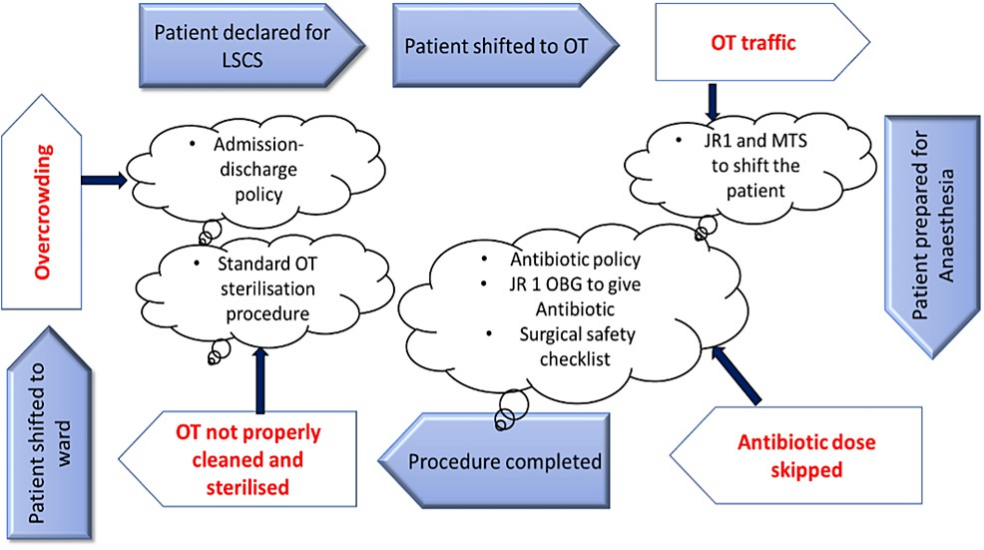
Process flow chart

The process flow chart was formulated to identify and solve the various problems (Figure 2).

The WHO recommends that routine antibiotic prophylaxis with a single dose of first-generation cephalosporin or penicillin should be given to all patients undergoing elective or emergency cesarean section [16]. Therefore, the most important step was to formulate the standard antibiotic policy and to ensure that antibiotic prophylaxis was given to all the patients within one hour of surgery. The standard operating procedure (SOP) would be designed for the OT where the resident (JR 1 OBG) on duty would be assigned the responsibility of administering the antibiotic. Injection cefazolin would be made available in the drug store of the hospital.

The reduction in the number of staff in the OT is an effective method for reducing environmental contamination near the surgical site. In OTs without laminar airflow (LAF), reducing door openings is an effective way to reduce environmental contamination and prevent SSI [17]. Various measures were discussed to decrease the OT traffic, which included designation and distribution of work and responsibilities of the staff, proper preoperative preparation of the patient, and a decrease in the frequency of shifts in the nursing and the anesthetic staff during surgery. The JR1 and MTS worker would shift the patient to OT, and a record would be made of the number of people in the OT during a procedure.

High bed occupancy rates have an impact on healthcare-associated infections (HCAIs) and methicillin-resistant Staphylococcus aureus (MRSA) [18]. There is a significant positive correlation between bed occupancy rates and the number of new MRSA cases identified [19]. The problem of overcrowding would be dealt with by a strict admission-discharge policy after consultation with the head of the neonatology unit.

Standard OT sterilization procedures and a surgical safety checklist would be prepared. Steps would be taken for the training of the staff in universal infection prevention practices (UIPP) and proper disposal of biomedical wastes.

The scrub sinks in OT would be converted to hands-free water control with digital timers, which would help in water conservation as well as decrease the risk of cross-contamination between users through the tap.

## Results

Strategy

Implementation of the change ideas

PDSA (Plan Do Study Act) Cycles

Table 2 shows the summary of the successive PDSA cycles and the lessons learned.

**Table 2:** PDSA cycles PDSA: Plan Do Study Act

S.NO	Change Idea Tested	When was it done	What was the result/ What did we learn
PDSA 1	SSI defined as wound infection / persistent fever ≥ 100°F for ≥ 48 hrs after the first 24 hours of surgery till 30 days	April 2019	Patients with fever from causes other than SSI were incorrectly diagnosed as SSI
PDSA 2	SSI was restricted to patients with wound infection till 30 days after surgery	July 2019	The modified definition was more clear and precise
PDSA 3	The surgical safety checklist displayed in all the maternity operation theaters	September 2019	Improving safety requires an understanding of the science of error and a consideration of human factors and systems failures
PDSA 4	SSI surveillance was modified from 30 days postoperative phase to the period till stitch removal	October 2019	Data collection became easier and more robust

PDSA 1: Surgical site infection was initially defined as purulent discharge from the wound and or persistent fever ≥100° F for ≥ 48 hours after the first 24 hours of surgery till 30 days. There was the strict implementation of policies with comprehensive training and monitoring (Table 3). The data were reviewed weekly.

**Table 3:** Developing and testing changes OT: operation theater; SOP: standard operating procedure; UIPP: universal infection prevention practices; BMW: biomedical waste; CS: cesarean section

Change Idea tested	When did we do this?	What was the result? What did we learn?
Review of OT sterilization log	2.4.2019	The OT was adequately sterilized after every surgery.
SOP for JR1 to administer antibiotic within 1 hour of surgery	15.4.2019	A prophylactic antibiotic was routinely given to patients undergoing CS.
SOP for workflow in the OT	15.4.2019	The OT traffic was decreased. The workflow in the OT was streamlined.
Training in UIPP and BMW management	29.4.2019	There was an improvement in UIPP and BMW management was organized.
Antibiotic policy document	13.05.2019	The antibiotic policy was formulated.
Admission discharge policy	20.05.2019	The problem of overcrowding was resolved to a great extent, which not only decreased the workload of the staff but also helped in decreasing infection.

PDSA 2: It was found that patients with fever due to other causes like malaria, dengue, tuberculosis, etc. were incorrectly documented as SSI. There was a need for the modification of the definition of SSI.

SSI was redefined and restricted to patients with wound infections occurring within 30 days of surgery. This decreased the errors occurring from incorrectly diagnosing patients with fever from other causes such as SSI.

The data were reviewed weekly.

PDSA 3:* *In spite of training and monitoring of the staff members and distribution of tasks, we were informed by the nurse in-charge of the maternity OT that some of the surgical checklists were filled retrospectively by the residents.

The WHO Surgical Safety Checklist has been developed after extensive consultation aiming to decrease errors and adverse events, and increase teamwork and communication in surgery. The Surgical Safety Checklist was displayed in all the maternity operation theaters to be read out aloud by the JR1 in OT to ensure the routine administration of prophylactic antibiotics and to decrease surgical and anesthetic errors.

PDSA 4: One particular difficulty for SSI surveillance in LMICs is that patients are often unwilling or unable to return to the hospital for multiple post-operative visits, particularly if the patient has no pressing symptoms. Also, should a problem occur following an operation, patients may then receive care from a healthcare provider different from the institution that performed the operation, meaning that the original surgeon may not be made aware of any potential surgical complications. A number of patients would get their stitches removed in the OPD making it even more difficult to track the patients.

To overcome the difficulty of tracking the postsurgical patients, SSI surveillance was modified from 30 days postoperative phase to the period till stitch removal. All the patients were directed to get their stitches removed in the postoperative ward only so that the follow-up with the patients would be easier.

The plan was adopted.

RESULTS

The baseline rate of SSI was 30% (Table 4).

**Table 4:** Rates of SSI in the patients operated by CS SSI: surgical site infections; CS: cesarean section

Month	Percentage of patients with SSI
March 2019 (baseline)	30%
April 2019	23%
May 2019	3%
June2019	15.67%
July 2019	5.25%
August2019	4.50%
September 2019	5.00%
October 2019	5.03%
November 2019	5.10%
December 2019	5.53%
January 2020	4.10%
February 2020	4.10%
March 2020	4.50%

In the first PDSA, due to the strict implementation of infection control measures and the dedicated efforts of all the team members, the rate of SSI started decreasing. In the month of May, the rate of SSI was 3%. There was an alarming increase in the next month to 15.6%. After further review and investigation, it was found that many cases of fever due to other causes like malaria, dengue, and tuberculosis were incorrectly documented as SSI.

In the second PDSA, SSI was strictly defined as a wound infection. The rate of SSI dropped to 5.25% in July.

In the third PDSA, the surgical safety checklists were displayed in OT, which benefitted the surgeons and anesthetists. It also decreased the workload of the residents who previously had to fill the checklists manually.

**Figure 3:**
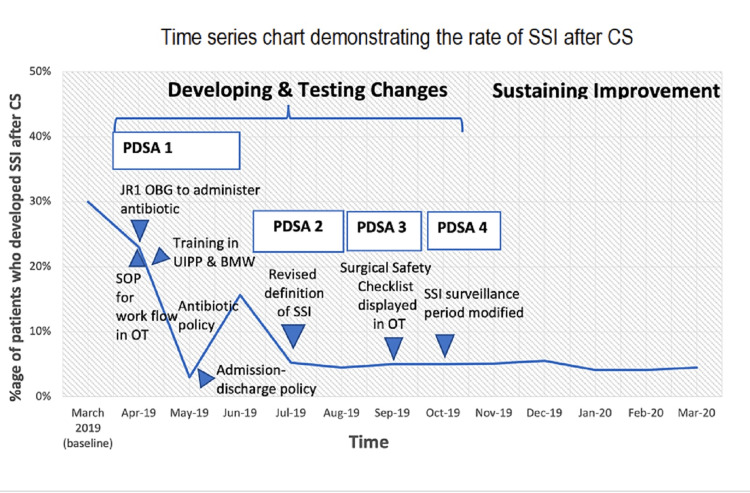
Results (time series chart/line graph)

In the fourth PDSA, the follow-up of the patients was reduced to the period till stitch removal, which made the data collection easier. In the third and the fourth PDSA, although the results were not changed in a significant way, the workload of the staff was decreased and the collection of data was simplified and vigorous. The rates of SSI have persistently remained around 5% (Figure 3).

## Discussion

The single most important risk factor for post-partum maternal infection is cesarean delivery [20]. Infectious complications following cesarean delivery include fever, wound infection, endometritis, bacteremia, other serious infection (including pelvic abscess, septic shock, necrotizing fasciitis, and septic pelvic vein thrombophlebitis), and urinary tract infection [20]. General principles for the prevention of any surgical infection include sound surgical technique, skin antisepsis, and antimicrobial prophylaxis [21].

Surgical site infection was quite prominent in our patients and a broad range of measures had to be adopted to decrease its incidence. A multidisciplinary team approach is important for quality improvement in various aspects. All team members have an important role to play. This QI project not only helped in decreasing the infection rates but also laid the foundation for other quality improvement projects.

In the first PDSA cycle, the major interventions were the formulation of an antibiotic policy, regular administration of prophylactic antibiotics before a skin incision, decreasing OT traffic, and resolving the problem of overcrowding.

The National Institute for Health and Care Excellence (NICE) and WHO guidelines on cesarean section recommend offering women prophylactic antibiotics at CS before a skin incision. For cesarean section, prophylactic antibiotics should be given prior to skin incision, rather than intraoperatively after umbilical cord clamping. The major problem before the QI project was that no one was specifically assigned the task of giving the antibiotic, which often led to skipping the dose or late administration.

WHO recommends that a single dose of first-generation cephalosporin or penicillin should be used in preference to other classes of antibiotics for antibiotic prophylaxis. The antibiotic policy was prepared and adapted by the other departments of the hospital as well. Injection cefazolin 2 gm IV was uniformly given to all the patients before the surgery, and it was made available in the hospital supply of drugs. While increased use of antimicrobial prophylaxis may be one factor in increasing antimicrobial resistance [22]. There are no data supporting the contention that the appropriate use of short-course antimicrobial prophylaxis will cause significant bacterial resistance nor evidence that a policy of antibiotic prophylaxis for cesarean section has harmful effects that outweigh its benefits, even in those women perceived to be at low risk [23]. Simple quality improvement methods have been demonstrated to improve adherence with the overall and timely administration of prophylaxis and reduce the infection rate [24].

Vicca reviewed the staffing levels and the incidence of new cases of MRSA over 19 months in an intensive care unit in a large tertiary referral center in the UK. The incidence of new cases of MRSA correlated with peaks in nursing staff workload and times of reduced nurse/patient ratios within the unit [25]. Overcrowding was a major problem in our hospital, as our hospital is a tertiary referral center and there was no admission discharge policy. The QI team formulated the admission discharge policy in collaboration with the department of pediatrics, which has helped in overcoming the problem of overcrowding in our wards to a great extent.

Surgical quality improvement projects should include the OT traffic pattern as an important parameter [26]. Lynch et al. reported that three common reasons for OR door openings were information issues (asking questions, checking on case status, or processing paperwork), personnel entering or leaving for breaks, and supply issues [27]. To decrease the OT traffic, protocols were made for shifting the patient and proper preoperative preparation of the patient. This not only decreased the rate of SSI but also streamlined the workflow in the OT.

The third PDSA involved the display of the surgical safety checklist in all the maternity OTs. The JR 1 OBG would read it out to the anesthetist, surgeon, and staff before starting the surgery. A surgical safety checklist is a simple tool designed to improve communication and teamwork by bringing together the surgeons, anesthesia providers, and nurses involved in care to confirm that critical safety measures are performed before, during, and after an operation. The WHO surgical safety checklist has been designed for routine use in operating theaters as a ‘standard operating procedure' [28]. The Harvard group has developed a number of checklists to be used during commonly encountered emergencies in theatre. Twelve ‘crisis checklists’ were developed after an appraisal of evidence and according to best practice [29]. The operating theater is the most common site for adverse surgical incidents, with errors occurring in nearly 15% of all patients globally, and some international studies suggest that surgery may be responsible for up to 1 million deaths every year, and an additional 7 million postoperative complications. The surgical safety checklist has been prepared by the QI team and displayed in every maternity OT, which has not only helped in decreasing the rate of infection but also in avoiding other surgical/anesthetic mishaps from occurring.

Hospital-associated infection (HAI) surveillance and timely feedback of results, including SSI surveillance, are strongly recommended by WHO as part of the core components of effective infection prevention and control (IPC) programs [30]. Every health facility should be committed to this in order to provide quality and safe health care and ensure that surveillance is not undertaken in isolation but is instead connected to other evidence-informed activities [30]. For feasibility reasons, in PDSA 4, post-discharge surveillance of SSI was limited to stitch removal, and all the patients were advised to get their stitches removed in the postoperative ward only. Low levels of follow-up of patients under surveillance is a concern, as there may be systematic differences in patients with complete follow-up data and those with incomplete data, which can lead to fundamental problems with surveillance results.

According to the WHO protocol for surgical site infection surveillance with a focus on settings with limited resources, there are various barriers and facilitating factors for a project [30].

The various barriers to the project are time and resource intensive (for example, increased workload), organizational ‘constipators’ - people who act as barriers and are reluctant to change, difficulty building the trust of staff who might feel they are being ‘watched’, absence of a patient safety culture that will support surveillance and improvement in the health care facility, objection to the need for dedicated surveillance resources, lack of capacity for data collection and local interpretation to allow for feedback, acceptance that the surveillance data are valid and reliable and that the theatre discipline is difficult to change [30].

The facilitating factors are influential and motivated individuals (surgeon, anesthetist, nursing champions, data collector, and manager), the involvement of a wide range of stakeholders, a sense of local ownership of the SSI data that can be raised among staff, and concrete leadership support. boundary spanners - individuals within a system that adopt the role of linking ‘networks’ (usually internal networks to external sources), peer-to-peer and inter-institution learning, clear action planning to ensure timely feedback, consistent and regular application of surveillance methods, and a method for data validation, particularly if data are used for benchmarking [30].

With the improvement in documentation and record keeping, we are able to monitor the infection rates in the patients so that there is no breach in the infection control measures. Prevention of SSI is possible for the most part.

Lessons and limitations

The lessons learned were that no plan is the best plan unless implemented and tested. Teamwork is essential. A multidisciplinary approach and the involvement of key stakeholders are necessary to address various problems. Meticulous record-keeping can be done but requires dedicated and motivated staff. Regular training and monitoring of the staff are essential for quality improvement. The staff nurses and sanitary workers should be actively involved in the projects and play a key role.

The limitations of the study were that there was no control group. As the study involved improvement in hospital policies, it was difficult to have a control group that would be deprived of the improvement measures. The postoperative surveillance period had to be shortened from 30 days to the period till stitch removal, as there was a loss of follow-up in some patients. A number of interventions were introduced in a short span of time, so it was difficult to interpret which process measure had a major role in decreasing the infection. Only wound infections were taken as SSI, so the patients with organ SSI-like endometritis were not included in the study.

## Conclusions

The rate of SSI has decreased in our patients. The antibiotic policy has been prepared and adapted by other departments of the hospital as well. We have improved our documentation and record-keeping. The admission discharge policy has been made in collaboration with the department of pediatrics, which has helped in overcoming the problem of overcrowding in our wards to a great extent. There is regular training of health care workers at all levels in universal infection prevention practices and BMW management. The workflow in the operation theater has been streamlined and the duties and responsibilities of the workers have been well-defined.
